# Presynaptic dopamine function measured with [^18^F]fluorodopa and L-DOPA effects on impulsive choice

**DOI:** 10.1038/s41598-019-54329-1

**Published:** 2019-11-29

**Authors:** Johannes Petzold, Ying Lee, Shakoor Pooseh, Liane Oehme, Bettina Beuthien-Baumann, Edythe D. London, Thomas Goschke, Michael N. Smolka

**Affiliations:** 1 Department of Psychiatry and Neuroimaging Center, Technische Universität Dresden, Dresden, Germany; 2 Freiburg Center for Data Analysis and Modeling, Albert-Ludwigs-Universität Freiburg, Freiburg, Germany; 3 Department of Nuclear Medicine, Technische Universität Dresden, Dresden, Germany; 4 Department of Psychiatry and Biobehavioral Sciences, Department of Molecular and Medical Pharmacology and the Brain Research Institute, University of California at Los Angeles, Los Angeles, CA USA; 5 Department of Psychology and Neuroimaging Center, Technische Universität Dresden, Dresden, Germany

**Keywords:** Human behaviour, Translational research, Decision

## Abstract

We previously reported that L-DOPA effects on reward-based decision-making in a randomized, placebo-controlled, double-blind, crossover study were consistent with an inverted U-shaped function whereby both low and high extremes of dopamine signaling are associated with high-impulsive choice. To test this hypothesis, we performed [^18^F]DOPA positron emission tomography in 60 of the 87 participants in that study, and measured the effective distribution volume ratio (EDVR) of [^18^F]DOPA influx rate to [^18^F]dopamine washout rate, an index of presynaptic dopaminergic function. Participants with higher baseline EDVR self-reported lower impulsivity, and discounted rewards as a function of delay more strongly after receiving L-DOPA, whereas the opposite was detected for those with lower baseline EDVR. Our findings support a relationship of striatal dopaminergic activity to trait impulsivity, and the view that there is a non-linear, possibly inverted U-shaped relationship of striatal dopaminergic function with delay discounting. Individuals with optimal dopamine signaling would become more impulsive when receiving dopamine-enhancing drugs, whereas those with suboptimal dopaminergic signaling would benefit and exhibit less impulsive choice. Consideration of differences in endogenous dopamine signaling and possibly also other neurotransmitter activity may be crucial to advance understanding of the neurobiochemical mechanisms of impulsive decision-making and related mental disorders.

## Introduction

Various mental health problems, including addictive behaviors^1,2^ and attention-deficit hyperactivity disorder (ADHD)^3^, feature impulsive decision-making, whereby individuals prefer smaller, immediate rewards over larger ones available after a delay (delay discounting) and probabilistic rewards over smaller, certain ones (risk-seeking for gains). Additional dimensions of decision-making encompass the propensity to overweight potential losses relative to equivalent gains (loss aversion) and to take risks to avoid certain losses (risk-seeking for losses). In part because dopamine-enhancing drugs are efficacious in the treatment of mental disorders (e.g., methylphenidate and amphetamine for ADHD) but also are abused^4,5^, an important role in decision-making has been attributed to dopamine. Since everyday life is full of choices involving trade-offs between reward magnitudes and probabilities or delays (e.g., picking the fastest line or best offer), one approach to delineate the role of dopamine is through pharmacological studies in healthy humans. Yet findings have been inconsistent^6^, with drugs that increase dopamine signaling as well as those that reduce it, both of which have been shown to boost and diminish impulsive choice^7^.

In a randomized, placebo-controlled, double-blind, crossover study, we recently found that L-DOPA had no main effect on impulsive decision-making, but had an effect on a probability discounting for gains task that was moderated by trait impulsivity as assessed with the Barratt Impulsiveness Scale (BIS-15)^7^. Moreover, changes in performance on delay discounting and mixed gambles tasks depended on trait impulsivity^7^. Participants with low impulsivity discounted rewards as a function of delay more strongly (measured by a delay discounting task), became more risk-seeking for gains (on a probability discounting for gains task) and more loss averse (on a mixed gambles task) after L-DOPA intake, whereas the opposite was exhibited by more-impulsive individuals^7^. In light of positron emission tomography (PET) studies that showed associations of impulsivity with pre- and postsynaptic neurochemical markers for dopamine signaling^8,9^, our results suggested an inverted U-shaped function whereby both low and high extremes of dopaminergic activity are linked to impulsive choice. Individuals with optimal dopamine signaling would get overdosed by dopamine-enhancing drugs, such as L-DOPA, and become more impulsive, whereas those with suboptimal dopaminergic signaling would make less impulsive choices.

Accumulating evidence supports the hypothesis that differences in dopamine signaling in striatal and prefrontal brain regions may underlie the individual variability in dopaminergic drug effects on cognitive control^10,11^. Findings obtained with a delay discounting task^12^ and the Balloon Analog Risk Task^13,14^, which involves sequential choices to pump a balloon to increase gains while risking explosion or to stop pumping to retain earnings, support this idea. Participants with higher trait impulsivity (presumed suboptimal dopaminergic signaling) showed greater effects of tolcapone, an inhibitor of the dopamine-degrading enzyme catechol-O-methyltransferase (COMT), to reduce discounting of rewards as a function of delay, as compared with less impulsive individuals^12^. An inverted U-shaped influence of dopamine, as indexed by [^18^F]fallypride PET^13^ or a composite score of functional polymorphisms across five genes^14^, has also been suggested for risky decision-making.

Here we extended prior work to determine if baseline dopaminergic activity affected the response to L-DOPA in several aspects of impulsive choice. As an index of presynaptic dopaminergic terminal function, we used [^18^F]DOPA PET, and determined the effective distribution volume ratio (EDVR), which is the ratio of [^18^F]DOPA influx rate to [^18^F]dopamine washout rate, and reflects the level of dopamine available for vesicular storage at steady state^15^. In a subset of 60 participants from our prior study^7^, we investigated whether the effects of L-DOPA on decision-making were related to intrinsic variations in striatal dopaminergic activity. We hypothesized that after L-DOPA administration, participants with lower striatal dopaminergic activity, as indexed by EDVR, would exhibit weaker delay discounting, reduced risk-seeking for gains and reduced loss aversion, whereas those with higher dopaminergic activity would show the opposite effects. As previous studies found no L-DOPA effects on risk-seeking for losses^7,16^, we predicted that baseline EDVR would not affect performance on a probability discounting for losses task. We further hypothesized that self-reported impulsivity (assessed with the BIS-15) would be negatively correlated with striatal dopaminergic activity.

## Methods

This work was part of the project “Dopaminergic Modulation of Meta-Control Parameters and the Stability-Flexibility Balance” within the Collaborative Research Center 940 “Volition and Cognitive Control: Mechanisms, Modulators and Dysfunctions” (www.sfb940.de). The project combined a pharmacological challenge of the dopamine system with questionnaires, behavioral tasks and neuroimaging. The methods were performed in accordance with relevant guidelines and regulations. All participants provided written informed consent as approved by the institutional review board of the Technische Universität Dresden (EK 44022012) and the Bundesamt für Strahlenschutz (Federal Office for Radiation Protection).

### Study procedure

We performed [^18^F]DOPA PET in 60 of the 87 participants from our previously published study^7^, where the flow of participants is described in detail. Briefly, we screened 1383 interested members of a representative population sample stratified by age and sex (N = 15778) using the following exclusion criteria: history of mental disorders except for nicotine dependence as per Structured Clinical Interview for DSM-IV^17^, history of major neurological disorders, visual acuity <0.8 with correction and contraindications to L-DOPA, magnetic resonance imaging (MRI) or PET. The detection of alcohol (breath-alcohol analysis on intervention visits; Alcotest 6510, Dräger, Lübeck, Germany) or other commonly used recreational drugs (urine test on first intervention visit; Kombi/DOA10-Schnelltest, Mahsan Diagnostika, Reinbek, Germany) led to exclusion from the study. Six hundred eleven eligible candidates completed a baseline visit, which included training on a decision-making battery and completion of the BIS-15 (range of scores: 15 to 60, higher scores reflect stronger impulsivity)^18^. The intervention visits followed a randomized, placebo-controlled, double-blind, crossover design in which Madopar (150 mg L-DOPA + 37.5 mg benserazide, a peripherally-acting DOPA decarboxylase inhibitor; Roche, Grenzach-Wyhlen, Germany) or a matched placebo was administered in tablet form followed by experiments that are not part of the present work. A booster dose of Madopar (75 mg L-DOPA + 18.75 mg benserazide) or a matched placebo was taken 100 min after the first dose and 50 min prior to the decision-making battery. All participants with sufficient quality of MRI and behavioral data from both intervention visits were invited to have an [^18^F]DOPA scan (mean days ± SD after the second intervention visit = 78.2 ± 51.90). Behavioral data evaluating L-DOPA effects on performance of decision-making tasks have been published recently^7^.

### Decision-making tasks

Four different facets of impulsive choice were assessed using a test battery that comprised four value-based decision-making tasks^19^ as previously described^7^: delay discounting (DD), probability discounting for gains (PDG), probability discounting for losses (PDL) and mixed gambles (MG). Each task provided binary offers, which were generated close to each individual’s estimated indifference point (corresponding to equal preference for both offers) using a Bayesian adaptive approach. The final estimation of k or λ values provided a quantitative index of impulsive choice on each task (see Table 1).

**Table 1:** Value functions for modeling and parameter estimations of decision-making tasks.

Decision-making task	Equation
Delay discounting	V = A/(1 + k D)
Probability discounting for gains	V = A/(1 + k [1 − p]/p)
Probability discounting for losses	V = A/(1 + k [1 − p]/p)
Mixed gambles	V = ½ (G − λ L)

V (subjective value of offer), A (amount of offer), k (discounting rate), D (length of delay), p (probability of winning or losing), G (amount of gain), λ (loss aversion parameter), L (amount of loss). Adapted from Pooseh *et al*.^19^.

The delay discounting task comprised 30 trials in which participants chose between a smaller, immediate or a larger, later reward (€5–30) to be received at varying delays (3, 7, 14, 31, 61, 180, 365 days), with higher k values indicating stronger discounting of delayed rewards. Thirty trials each for PDG and PDL presented choices between a sure gain or loss and winning or losing a bigger amount of money (€5–30) with varying probabilities (2/3, 1/2, 1/3, 1/4, 1/5). Stronger discounting of probabilities is reflected by higher k values, indicating risk aversion in PDG (preference for certain over probabilistic gains) but risk-seeking in PDL (preference for probabilistic over certain losses). Forty gambles with a 50% chance of winning (€1–40) or losing (€5–20) were offered in MG, with higher λ values reflecting stronger loss aversion (tendency to reject gambles). Participants were not informed about probabilistic outcomes, but were instructed that at the end of each visit one trial per task would be selected randomly and paid according to the given choice to ensure realistic behavior.

### Positron emission tomography

Imaging data were acquired and processed as described previously^20^. Briefly, participants were asked to abstain from protein-containing breakfasts before a 4-hour [^18^F]DOPA scan (Ingenuity TF PET/MR system, Philips Healthcare, Cleveland, OH, USA) at rest. Carbidopa (150 mg in tablet form, a peripherally-acting DOPA decarboxylase inhibitor; Amerigen Pharmaceuticals, Lyndhurst, NJ, USA) was administered 60 min prior to intravenous [^18^F]DOPA injection to increase striatal activity and the signal-to-noise ratio^21^. Striatal dopamine availability at steady state was calculated by estimating EDVR on a voxel-wise basis by graphical analysis, and averaging the value within the whole striatum. In exploratory analyses, striatal subregions (caudate nucleus, putamen and ventral striatum) were evaluated.

### Statistical analyses

We used SPSS Statistics 25 (IBM, Armonk, NY, USA) and assumed two-tailed significance at p < 0.05 for all analyses. Histograms and normal quantile-quantile plots were used to judge normality. To meet the assumptions of parametric testing, k and λ values were log-transformed. All correlations are reported as Pearson’s r.

To address whether EDVR interacted with L-DOPA-induced changes in impulsive choice, we determined partial correlations between whole-striatal EDVR and differences of performance on decision-making tasks between drug conditions (Δlog k/λ = log k/λ_L-DOPA_ − log k/λ_Placebo_). We controlled for drug order because some participants received placebo and others L-DOPA first (crossover design). In keeping with our previous analyses^7^ and studies that observed bodyweight-dependent effects of L-DOPA^16,22^, we also controlled for weight and then examined correlations of interest restricted to low-weight participants (≤80.5 kg at L-DOPA visit). Exploratory analyses were carried out to investigate results in striatal subregions: caudate nucleus, putamen and ventral striatum.

We evaluated correlations between EDVR and trait impulsivity (BIS-15 total score) as well as decision-making performance after participants had taken placebo (log k/λ_Placebo_). To check if correlations were confounded, we reassessed all analyses while controlling for potential nuisance variables (age^23,24^, sex^24^, season^25^) that were significantly associated with EDVR in the whole striatum. To interpret non-significant results, we conducted a post-hoc power analysis (p < 0.05, one-tailed, N = 60, correlation coefficient of null hypothesis = 0) using G*Power Version 3.1.9.4 (www.gpower.hhu.de)^26^.

## Results

The 60 participants (mean age ± SD at PET visit = 36.4 ± 3.84; 49 males, 11 females) reported low to moderate impulsivity (mean BIS-15 ± SD at baseline = 29.9 ± 5.23). The mean (±SD) EDVR for the whole striatum and striatal subregions caudate nucleus, putamen and ventral striatum were 1.45 (±0.18), 1.13 (±0.18), 1.74 (±0.21) and 1.26 (±0.18), respectively.

**Figure 1:**
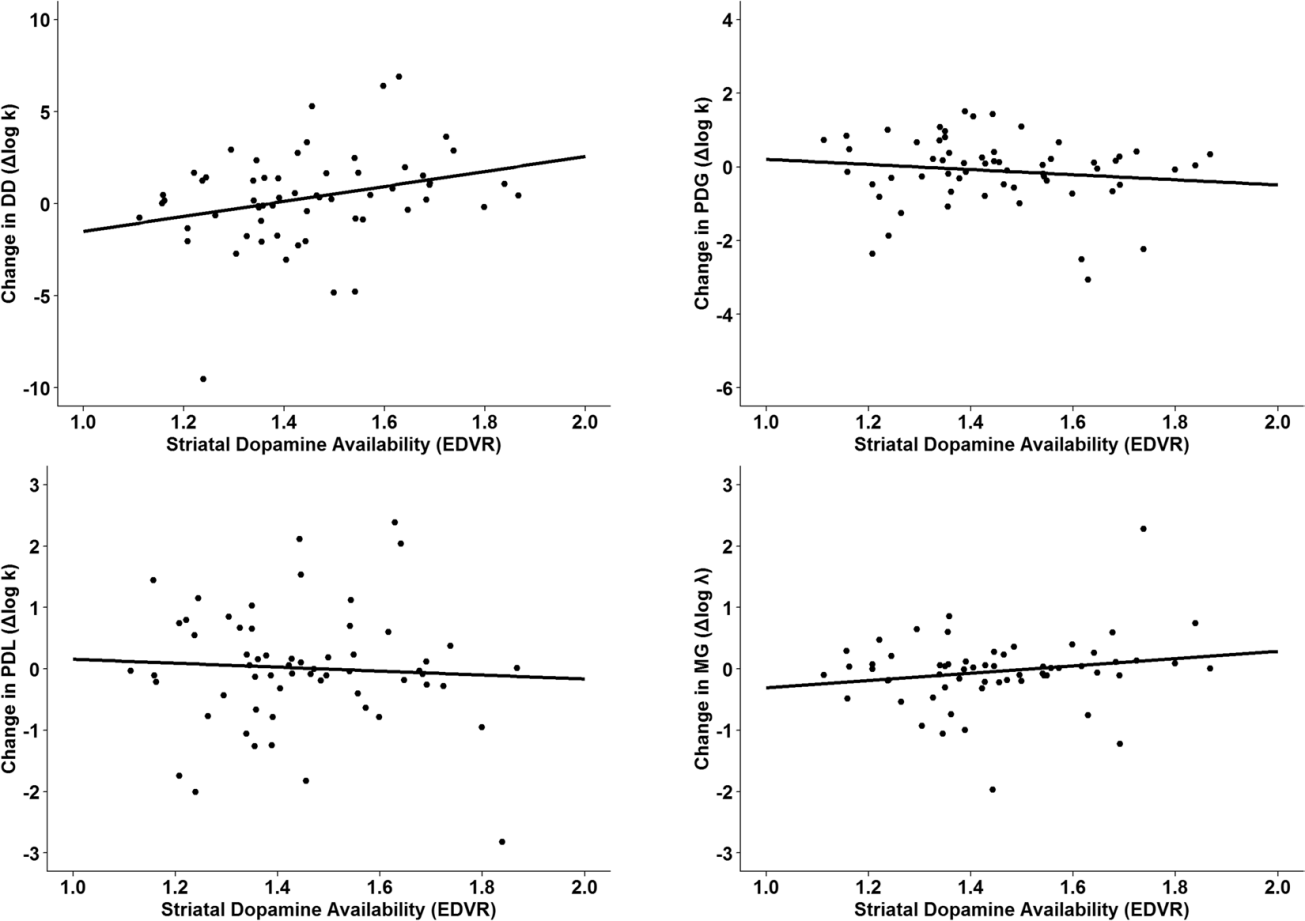
Relationship between change in decision-making tasks by L-DOPA (Δlog k/λ = log k/λ_L-DOPA_ − log k/λ_Placebo_) and presynaptic dopaminergic function in the whole striatum. DD (delay discounting), PDG (probability discounting for gains), PDL (probability discounting for losses), MG (mixed gambles).

Whole-striatal EDVR was correlated with L-DOPA-induced change (Δlog k/λ = log k/λ_L-DOPA_ − log k/λ_Placebo_) in delay-discounting behavior (Δlog k_DD_) as hypothesized (r = 0.281, p = 0.031; see Fig. 1). Post-hoc analyses revealed that the correlation was driven by the putamen (r = 0.301, p = 0.020), whereas correlations with the caudate nucleus (r = 0.245, p = 0.061) and the ventral striatum (r = 0.080, p = 0.548) did not reach significance. L-DOPA-induced changes in risk-seeking for gains (Δlog k_PDG_) and loss aversion (Δlog λ_MG_) were not significantly related to EDVR (see Fig. 1, Table 2).

**Table 2:** Pearson’s partial correlations between striatal EDVR and L-DOPA-induced changes in impulsive choice (Δlog k/λ = log k/λ_L-DOPA_ − log k/λ_Placebo_).

	Controlled for	Whole striatum	Caudate nucleus	Putamen	Ventral striatum
Delay discounting(Δlog k_DD_)	Drug order	0.281 (0.031)*	0.245 (0.061)	0.301 (0.020)*	0.080 (0.548)
	Drug order + season	0.283 (0.031)*	0.245 (0.064)	0.304 (0.020)*	0.075 (0.576)
	Drug order + season + body weight	0.326 (0.013)*	0.280 (0.035)*	0.344 (0.009)*	0.122 (0.366)
Risk-seeking for gains(Δlog k_PDG_)	Drug order	−0.139 (0.294)	−0.136 (0.306)	−0.121 (0.361)	−0.039 (0.772)
	Drug order + season	−0.162 (0.226)	−0.156 (0.241)	−0.143 (0.283)	−0.050 (0.708)
	Drug order + season + body weight	−0.106 (0.432)	−0.109 (0.420)	−0.091 (0.501)	0.033 (0.808)
Risk-seeking for losses(Δlog k_PDL_)	Drug order	−0.064 (0.631)	−0.074 (0.578)	−0.051 (0.704)	−0.043 (0.746)
	Drug order + season	0.002 (0.988)	−0.013 (0.925)	0.017 (0.900)	0.000 (0.997)
	Drug order + season + body weight	0.039 (0.771)	0.019 (0.890)	0.052 (0.701)	0.050 (0.711)
Loss aversion(Δlog λ_MG_)	Drug order	0.184 (0.163)	0.209 (0.112)	0.159 (0.230)	0.139 (0.293)
	Drug order + season	0.214 (0.107)	0.238 (0.072)	0.188 (0.158)	0.156 (0.241)
	Drug order + season + body weight	0.240 (0.072)	0.260 (0.051)	0.210 (0.116)	0.190 (0.157)

Drug order was controlled for as participants received either placebo or L-DOPA first (crossover design). Season was grouped in spring/summer and fall/winter. N = 60. P values are in parentheses. *p < 0.05.

We considered age^23,24^, sex^24^ and season^25^ as nuisance variables because of evidence that they may affect central dopamine. While neither sex (r = −0.061, p = 0.645) nor age (r = 0.097, p = 0.460) significantly influenced EDVR, we found a significant association between season (grouped in spring/summer and fall/winter) and EDVR in the whole striatum (r = 0.283, p = 0.028). Absolute EDVR values (mean ± SD) of participants (n = 25) who underwent the [^18^F]DOPA scan in the fall-winter period (1.51 ± 0.17) were higher than those of participants (n = 35) examined in spring or summer (1.40 ± 0.18). Controlling for season using partial-correlation analyses however did not substantially impact L-DOPA-induced changes in impulsive choice (see Table 2). After including weight as another nuisance variable due to possible bodyweight-dependent effects of L-DOPA^7,16,22^, EDVR in the whole striatum (r = 0.240, p = 0.072) tended to predict L-DOPA-induced change in loss aversion (Δlog λ_MG_, see Table 2). Using our previously applied body weight threshold (≤80.5 kg)^7^, we restricted correlational analyses to 30 low-weight participants and obtained similar results, but the correlation between change in loss aversion (Δlog λ_MG_) and EDVR in the ventral striatum became significant when controlling for seasonal variation (r = 0.381, p = 0.045).

BIS-15 was inversely related to EDVR in the whole striatum as predicted (r = −0.268, p = 0.040) and also in the ventral striatum (r = −0.261, p = 0.046) after controlling for seasonal variation. Table 3 displays the correlation matrix between EDVR and decision-making performance under placebo, with only the negative correlation between delay-discounting behavior and putaminal EDVR being significant (with and without season included as nuisance variable).

**Table 3:** Pearson’s correlations between striatal EDVR and impulsive choice under placebo (log k/λ_Placebo_). Season was grouped in spring/summer and fall/winter. N = 60. P values are in parentheses. *p < 0.05.

	Controlled for	Whole striatum	Caudate nucleus	Putamen	Ventral striatum
BIS-15	Uncontrolled	−0.238 (0.068)	−0.158 (0.228)	−0.214 (0.101)	−0.245 (0.059)
	Season	−0.268 (0.040)*	−0.182 (0.168)	−0.243 (0.063)	−0.261 (0.046)*
Delay discounting(log k_DD_)	Uncontrolled	−0.231 (0.075)	−0.088 (0.503)	−0.292 (0.024)*	−0.119 (0.365)
	Season	−0.200 (0.130)	−0.051 (0.699)	−0.264 (0.044)*	−0.095 (0.473)
Risk-seeking for gains(log k_PDG_)	Uncontrolled	0.134 (0.306)	0.116 (0.376)	0.122 (0.352)	−0.004 (0.974)
	Season	0.100 (0.452)	0.083 (0.532)	0.087 (0.513)	−0.030 (0.821)
Risk-seeking for losses(log k_PDL_)	Uncontrolled	−0.122 (0.353)	−0.199 (0.127)	−0.114 (0.388)	−0.065 (0.621)
	Season	−0.132 (0.320)	−0.211 (0.109)	−0.123 (0.354)	−0.069 (0.605)
Loss aversion(log λ_MG_)	Uncontrolled	−0.080 (0.542)	−0.073 (0.579)	−0.073 (0.579)	−0.088 (0.502)
	Season	−0.127 (0.336)	−0.116 (0.380)	−0.120 (0.364)	−0.117 (0.377)

A post-hoc power analysis revealed that our whole sample (N = 60) had sufficient power (0.763) to detect correlations of medium (r = 0.3), but not of smaller effect sizes (power = 0.460 for r = 0.2, power = 0.189 for r = 0.1). To avoid increasing the already high type II error probability, we decided against the correction for multiple comparisons in the analyses involving correlations with test performance.

## Discussion

Our objective was to investigate whether individual variations in striatal EDVR, an index of presynaptic dopaminergic function, would be related to L-DOPA-induced changes in impulsive choice. As hypothesized, participants who reported low impulsivity exhibited higher EDVR, and discounted reward as a function of delay more strongly after L-DOPA administration, whereas lower EDVR was exhibited by more-impulsive individuals (BIS-15), who exhibited less delay discounting after receiving L-DOPA. Participants with higher EDVR also tended to be more risk-seeking for gains and loss averse after L-DOPA intake, while those with lower baseline EDVR behaved in an opposite way, but this effect reached statistical significance only for the relation between loss aversion and the EDVR in the ventral striatum of low-weight participants, possibly due to a stronger L-DOPA effect because dose was not adjusted for body weight. As hypothesized, differential baseline dopamine availability did not moderate L-DOPA-induced changes in risk-seeking for losses.

Our study adds to the mounting evidence that the relationship between dopamine signaling and cognitive function is not linear, but follows an inverted U-shaped curve^10,11^. This theory implies that contrasting effects on cognition may occur depending on how dopaminergic drugs shift dopaminergic activity on this curve. Such an influence of dopaminergic function has also been assumed in a study in which tolcapone, an inhibitor of the dopamine-degrading enzyme catechol-O-methyltransferase (COMT), reduced delay discounting in participants with high impulsivity, whereas low-impulsive participants (presumed optimal dopaminergic signaling) exhibited smaller declines or enhancements in delay discounting^12^. Of note, tolcapone may preferentially increase frontal dopamine as the dopamine transporter (DAT), which inactivates dopamine by reuptake, is considerably less abundant in frontal than other brain regions^10,12^. The data from that study indicated a circuit mechanism for dopamine effects since delay discounting varied with tolcapone-induced changes in the left ventral putamen and the frontostriatal connectivity^12^.

Our finding that low EDVR in the putamen but not in the caudate nucleus or ventral striatum was related to delay discounting (under placebo) is consistent with that of a PET study in 16 healthy adults using [^18^F]fluoro-meta-tyrosine as radiotracer to measure dopamine synthesis capacity^27^. The independence of the negative association between putaminal PET signal and delay discounting of COMT genotype^27^ endorses the hypothesis that individual variability of dopaminergic activity in both the frontal cortex and putamen modulates delay discounting. An inverted U-shaped dopamine function has also been concluded from associations between delay-discounting behavior and left caudate synthesis capacity using [^18^F]DOPA in patients with Parkinson’s disease and ventral striatal D2/D3 dopamine receptor availability using [^11^C]raclopride in pathological gamblers^28^. The latter adds to the literature that links delay discounting to dopaminergic activity in the ventral striatum^11,29^, whereby distinct and partly opponent mechanisms in impulsivity have been attributed to its subregions the core and shell of the nucleus accumbens^27,29^. The low spatial resolution of PET, which did not allow us to disentangle these subregions, may have caused net cancellation of effects as speculated previously^27^.

Further support for the inverted U-shaped concept comes from studies on risky decision-making applying the Balloon Analogue Risk Task and assessing striatal dopaminergic signaling using a composite score of functional polymorphisms across five genes encoding dopamine receptors (D2, D3, D4), DAT and COMT^14^, or striatal D2/D3 availability using [^18^F]fallypride PET^13^. In line with these findings, our data show a tendency of caudate presynaptic dopaminergic activity predicting L-DOPA-induced changes in delay discounting and trends of positive association between change in loss aversion and dopamine in the caudate nucleus and the ventral striatum. L-DOPA-induced changes in risk-seeking for gains were inversely correlated to striatal dopamine as predicted, albeit far from reaching statistical significance. Although our study lacks power to detect correlations of smaller effect sizes, the literature indicates that different brain networks, neurotransmitters and possibly a family of functions (e.g., biphasic, sigmoidal, exponential) are differently involved in distinct facets of impulsive choice^29,30^. Consistent with our previous work^7^ and other reports^16,31^, we found no evidence for dopaminergic neurotransmission affecting risk-seeking for losses. In fact, serotonergic tone may modulate the sensitivity towards valuation of losses^31^.

Research findings suggest that greater trait impulsivity as measured by BIS is characterized by lower dopamine signaling^8,9^, with contrasting data reported in small-sample studies limited to BIS subscales^32,33^. Higher BIS-11 impulsivity and lower striatal D2/D3 availability were found in recently abstinent methamphetamine-dependent individuals compared to healthy controls with BIS-11 related to D2/D3 receptor availability across both groups^9^. Higher BIS-11 scores also correlated with higher striatal DAT availability (consistent with lower dopaminergic tone) in 38 healthy men^8^. Our data based on a different PET tracer in a large sample of healthy men and women support these results, which may be reconciled with an inverted U-shaped function, assuming that the relation between dopamine (increasing from left to right on x-axis) and BIS (decreasing from bottom to top on y-axis) reflects the ascending limb while the descending limb does not appear as acutely overdosed individuals were not tested.

In conclusion, this study corroborates and extends previous findings demonstrating the importance of dopamine signaling in impulsivity by providing further insights into the biological underpinnings of impulsive decision-making. Our data indicate that lower dopaminergic activity in the striatum is a correlate of stronger trait impulsivity and delay discounting. They also suggest that the latter can be modulated by dopaminergic drugs depending on individual variations in the striatal dopamine system, possibly following an inverted U-shaped curve. Whereas substantial evidence identifies dopamine as a crucial determinant of delay-discounting behavior, our results highlight that distinct dimensions of impulsive decision-making seem to be differently regulated. Yet our findings must be interpreted with caution since we did not correct for multiple comparisons in the analyses involving correlations with test performance.

Appreciation of variations in endogenous dopamine availability and the complexity of different neurotransmitter and brain systems implicated in impulsivity are therefore needed in future pharmacological studies. The identification of mutual as well as unique neural underpinnings of different facets of impulsive choice will advance our understanding of impulsivity and may also offer new insights into related mental health problems. In fact, impulsive choice has already been considered as a promising target for the development of more effective interventions across mental disorders^34,35^.

## Data Availability

All data generated and analyzed during this study are available from the corresponding author on reasonable request.
